# Sepsis-related hospitalisation risk in preterm-born and term-born children and adolescents: a nationwide registry study

**DOI:** 10.1016/j.eclinm.2026.104295

**Published:** 2026-11-03

**Authors:** Jonas Valand, Sara Marie Nilsen, Andreas Asheim, Markus Haug, Henrik Døllner, Tormod Rogne, Erik Solligård, Kari Risnes

**Affiliations:** a Faculty of Medicine and Health Sciences, Department of Clinical and Molecular Medicine, Norwegian University of Science and Technology, Trondheim, Norway; b Children's Clinic, St. Olavs University Hospital, Trondheim, Norway; c Department of Chronic Disease Epidemiology, Yale School of Public Health, New Haven, CT, USA; d Department of Community Medicine and Global Health, University of Oslo, Oslo, Norway; e Department of Circulation and Medical Imaging, Mid-Norway Sepsis Research Center, Norwegian University of Science and Technology and St Olavs University Hospital, Trondheim, Norway

**Keywords:** Sepsis, Children, Adolescents, Gestational age, Preterm, Infectious diseases

## Abstract

**Background:**

The burden of severe infections in children remains inadequately characterised. Preterm birth is a risk factor for neonatal sepsis, but whether this risk persists beyond the neonatal period is unclear. We examined the risk of sepsis-related hospitalisation during childhood and adolescence in term-born and preterm-born children.

**Methods:**

Norwegian nationwide birth registry data (Jan 1, 1992–Dec 31, 2021) were linked with hospital discharge data (Jan 1, 2008–Dec 31, 2021). Using a standardised definition, sepsis-related hospitalisations were classified as explicit (sepsis diagnosis code) or implicit (infection plus organ dysfunction diagnosis codes). We estimated rate ratios (RRs) using adjusted negative binomial regression and sibling-controlled odds ratios (ORs) using conditional logistic regression by completed weeks of gestation (23–27, 28–31, 32–33, 34–36, 37–38, and 39–41 weeks), stratified by age (3–11 months, 1–5 years, and 6–15 years).

**Findings:**

Following 1,672,009 births, 13,158 sepsis episodes were available in the data. Hospitalisation rates for sepsis-related admissions were highest at ages 3–11 months (84.4 per 1000 person-years [95% CI 62.2–106.7] for 23–27 weeks gestation and 3.2 per 1000 person-years [95% CI 3.0–3.4] for 39–41 weeks gestation). Compared with term-born children, rates of sepsis-related hospitalisations increased by lower gestational age groups in both population and sibling analyses. Comparing extremely preterm versus term-born children, RRs were 26.5 (95% CI 20.2–34.7) at ages 3–11 months, 14.8 (11.2–19.6) at ages 1–5 years, and 6.7 (3.9–11.4) at ages 6–15 years. Sibling ORs were 24.9 (10.8–57.4), 29.7 (11.3–77.8), and 3.1 (1.2–8.0), respectively.

**Interpretation:**

Lower gestational age was robustly associated with an increased risk of sepsis-related hospitalisation throughout childhood and adolescence, supporting that low gestational age at birth should be recognised as a persistent risk factor for severe infections beyond the neonatal period.

**Funding:**

The Regional Cooperation Body for Education, Research and Innovation between the Central Norway Regional Health Authority (Helse Midt-Norge RHF) and the universities and university colleges in the region (phd funding for Jonas Valand) and the joint research committee of St. Olavs University Hospital and Faculty of Medicine and Health Sciences, Norwegian University of Science and Technology (grant no. 2024-38542).

**Research in context**

**Evidence before this study**

Preterm birth is an established risk factor for neonatal sepsis and early childhood infections, but whether this increased vulnerability to severe, life-threatening systemic responses persists into late childhood and adolescence remains poorly understood. We searched PubMed using the keywords (“gestational age”, “preterm”, or “premature”) AND (“sepsis” or “septicaemia”) AND (“admission”, “hospitalisation”, or “inpatient”) for articles in any language from its inception up to 11 May 2026. The search returned 1241 articles, of which many concerned neonatal sepsis risk. We did, however, not identify any studies that assessed the risk of post-neonatal sepsis with gestational age as exposure.

**Added value of this study**

Using a comprehensive nationwide cohort of over 1.6 million individuals followed for up to 15 years, this study provides precise, age-specific incidence estimates for sepsis-related hospitalisations across all gestational ages. We identified a clear dose–response relationship, demonstrating that shorter gestational age at birth is associated with a progressively increased risk of admission for severe infections. By using robust sibling-controlled analyses, we confirmed that this persistent, pronounced excess risk among children born preterm extends from early childhood through mid-adolescence and therefore is unlikely to be fully explained by shared familial or environmental factors.

**Implications of all the available evidence**

The elevated risk of severe infections and sepsis-related hospitalisation in individuals born preterm is not confined to the neonatal period but represents a long-lasting vulnerability. Low gestational age at birth should be formally recognised as an ongoing risk factor for severe post-neonatal infections. These findings underscore the need to prioritise infection prevention measures, tailor vaccination programmes, and maintain high clinical vigilance for severe infections in children and adolescents born preterm.

## Introduction

Preterm birth is a recognised risk factor for infectious diseases in children.^1^, ^2^ Evidence suggests that this increased vulnerability may persist into adulthood, exhibiting a dose–response relationship, with lower gestational age at birth associated with higher subsequent risk of infection.^3^, ^4^ Compared with term-born individuals, those born extremely preterm have the highest risk estimates, up to tenfold for infection-related hospitalisations, while even those born early term exhibit elevated risk relative to full-term births. Although large cohort studies have elucidated the long-term adverse health outcomes of preterm infants, their susceptibility to severe infections and sepsis beyond the neonatal period remains poorly understood.^5^, ^6^, ^7^

Sepsis, a severe systemic response to infections, accounts for approximately half of deaths in children under 5 years globally and can result in long-term health sequelae.^8^, ^9^, ^10^ In sepsis, infection triggers a dysregulated immune response that may cause life-threatening organ dysfunction.^11^, ^12^ Children with underlying conditions are disproportionately affected.^13^, ^14^, ^15^

Immunological studies indicate that preterm infants exhibit lower levels of passively acquired maternal antibodies.^16^, ^17^ T cell activation responses are impaired in preterm-born children during the initial weeks after birth but generally show signs of recovery over time.^18^ Whether these early immunological perturbations contribute to a prolonged inflammatory state and dysregulated immune responses beyond the neonatal period remains unclear.^19^

Previous estimates of infection risk in preterm-born children have key limitations. Risk estimates may reflect a lower threshold for hospitalisation in this population rather than the true disease burden. Furthermore, few studies have analysed gestational age as a continuous or non-linear variable, potentially obscuring nuances in risk. Consequently, the burden of severe infections beyond the neonatal period remains inadequately characterised. By focussing specifically on sepsis-related hospitalisation, using established definitions based on hospital discharge data,^9^, ^13^ this study aimed to provide more precise and less biased risk estimates.

Using a near-complete Norwegian population cohort, we examined the risk of sepsis-related hospitalisation in children and adolescents, stratified by gestational age at birth. We further assessed whether these associations are explained by genetic and social factors shared by siblings.

## Methods

### Study design and data

We used data from the Medical Birth Registry of Norway (MBRN) and included all births between Jan 1, 1992, and Dec 31, 2021. MBRN records were linked to hospital discharge data from the Norwegian Patient Registry from Jan 1, 2008, to Dec 31, 2021. Dates of death and emigration were obtained from the Norwegian Cause of Death Registry.

The unique 11-digit personal identity number assigned to all Norwegians at birth or upon immigration enabled precise linkage of data across multiple sources. The study population was defined through the MBRN, and a project-specific participant identifier was created. Data access and linkage were approved by Heath Data Norway (H-1338-E). Researchers accessed only de-identified data. Information on the registries, variables and access to data for research can be found at https://helsedata.no/en/.

The study was based on all births registered in Norway between Jan 1, 1992, and Dec 31, 2021, in the Medical Birth Registry of Norway. Individuals were eligible for inclusion if they had a valid personal identifier and were live born; information on gestational age and birthweight; gestational age between 23 weeks and 44 weeks; birthweight between 300 g and 6500 g; birthweight-for-gestational-age values within plausible ranges; maternal age of 55 years or younger at delivery; and follow-up time available between age 3 months and 15 years during the study period (Jan 1, 2008–Dec 31, 2021).

### Ethics

The study was approved by the Regional Committee for Medical and Health Research Ethics (REK-Midt, 2018/32). Although informed consent is not required for inclusion in national registries, individuals retain the right to request the exclusion of their data from these registries.

### Exposure

Gestational age in days was obtained from MBRN, based primarily on routine ultrasound examinations. If ultrasound data were unavailable, gestational age was calculated from the date of the last menstrual period or, in cases of assisted reproductive technology (ART), from the date of conception. Gestational age was converted into completed weeks at birth and categorised as extremely preterm (23–27 weeks), very preterm (28–31 weeks), moderately preterm (32–33 weeks), late preterm (34–36 weeks), early term (37–38 weeks), full-term (39–41 weeks), and post-term (42–44 weeks).^20^

### Outcomes

Using a standardised definition, sepsis-related hospitalisations were classified as implicit sepsis (infection plus organ dysfunction diagnosis codes) as the primary outcome. In secondary analyses, explicit sepsis (sepsis diagnosis code) was assessed. Implicit sepsis was defined as a combination of infection as the main diagnosis and any acute organ dysfunction code (eTable S1). Explicit sepsis was identified as a hospital admission with a specific International Classification of Diseases, 10th Revision (ICD-10) code for sepsis as the main diagnosis. Several adult studies have previously revealed low sensitivity (around 35%) but high specificity (around 98%) for sepsis using explicit criteria based on hospital discharge data.^21^ One paediatric reported sensitivity and specificity values of explicit coding to 35% and 95%, and for an implicit strategy to be 65% and 87%, respectively.^22^ Sepsis episodes were also categorised by organ system aetiology based on the specific primary infection codes. The current sepsis definition was introduced for adults in 2016 and updated for paediatrics in 2024 to align with these changes, including modern physiological criteria such as the PHOENIX consensus definition.^11^, ^12^

### Statistics

Statistical analyses were performed using Stata version 18.0.^23^ The association between gestational age at birth and sepsis-related hospitalisation was quantified by calculating incidence rates and incidence rate ratios (RRs) using negative binomial regression models. Incidence rates were calculated using person-years at risk, excluding time spent in the hospital. Each hospitalisation contributed a single event; however, hospital transfers and readmissions belonging to the same illness episode were treated as a single continuous event. Time at risk was defined as the total duration from 3 months to 15 years of age during the period 1 Jan 2008–31 Dec 2021, accounting for delayed entry (left-truncation) for children born before 1 Jan 2008, or until the end of follow-up, emigration, or death, whichever occurred first. The observation period was further stratified into three specific age intervals: 3–11 months (including the full 11th month), 1–5 years (including the full 5th year), and 6–15 years (including the full 15th year).

The main analysis was adjusted for month and year of birth, sex, maternal age, parity, immigration status (defined as maternal country of birth, serving as a proxy for structural and social determinants of health), and the method used to determine gestational age (ultrasound, last menstruation, or ART). Supplemental analyses were performed by separately adding to the main model adjustment for birth weight Z-scores as indicator of foetal growth and maternal education as indicator of socioeconomic status. Multiple births were retained, with each infant treated as an independent observation. To focus on post-neonatal infections, the neonatal period was excluded, and the lower age limit for inclusion was set at 3 months. Chronological age from birth, rather than corrected age for prematurity, was used to define age at follow-up.

Given that the Rudd definition encompasses both explicit and implicit sepsis diagnoses (infection plus organ failure),^9^ sensitivity analyses were conducted to test the robustness of this definition. To evaluate the effect using only the highly specific definition, a sensitivity analysis was conducted where explicit sepsis was analysed alone. To isolate the risk of severe infection requiring hospitalisation from the known risk of severe lung disease in preterm infants,^3^, ^24^ a pre-specified sensitivity analysis was conducted excluding respiratory failure as a component of sepsis among patients with respiratory tract infections (RTIs). Additionally, to examine the potential impact of perinatal inflammatory processes on immune system development, assuming that spontaneous preterm vaginal deliveries are often inflammation-driven, the main analyses were repeated stratified by medically indicated births (e.g., inductions or planned caesarean deliveries). Sensitivity analyses also included additional adjustment for maternal education and birth weight z scores and after excluding congenital malformations.

Sibling analyses were conducted using conditional logistic regression to control for shared familial factors. Comparisons were made between siblings who differed in both gestational age at birth and sepsis outcome within each follow-up age category, thereby helping to determine whether any observed associations were attributable to gestational age, rather than unmeasured familial characteristics shared by siblings.^25^ Analyses were stratified by sibling sets in which at least two children from the same mother contributed person-time within a given follow-up age band (3–11 months, 1–5 years, and 6–15 years). For each age interval, a binary outcome was defined as having ≥1 hospital admission for sepsis during that interval. Conditional logistic regression was then performed within mother-specific strata, with results presented as within-family odds ratios (ORs) for gestational age categories. This approach inherently accounts for genetic and environmental factors shared among siblings. However, because it is based on binary outcomes rather than person-time, effect estimates are not directly comparable to the rate ratios from the main analyses. As in the main analyses, sibling analyses were adjusted for sex, month and year of birth, maternal age, maternal birth country, parity, and method used to determine gestational age.

The protocol for the study originally included analyses in age groups from neonates to adults and a replication in Swedish data, as shown in eAppendix 1. The adult analyses and replication in Swedish data have been postponed for a separate publication. The neonatal analyses were not performed due to limitations in data granularity for post-partum hospital stays.

This study was conducted and reported in accordance with the Strengthening the Reporting of Observational Studies in Epidemiology (STROBE) guidelines.

### Role of the funding source

The study was funded to NTNU by Central Norway Regional Health Authority, St. Olavs University Hospital and The joint research committee of St. Olavs University Hospital and Faculty of Medicine and Health Sciences, Norwegian University of Science and Technology. The funders of the study had no role in study design, data collection, data analysis, data interpretation, or writing of the report.

## Results

1,785,117 births were identified in the Medical Birth Registry of Norway (Fig. 1). Of these, 76,079 (4.3%) were excluded because of missing or implausible values in eligibility variables, and a further 13,224 (0.7%) were excluded due having no follow up time in the observation period (e.g., death or emigration before 3 months of age or before the 2008 study start). The final study population comprised 1,672,009 children and adolescents. Baseline characteristics are presented in Fig. 2. We identified 13,158 total hospital admissions that met the implicit sepsis criteria during follow-up, of which 2660 (20.2%) were coded as explicit sepsis diagnoses. By age group, 854,965 children contributed observation time in the 3–11 months interval (2079 experienced ≥1 sepsis episode), 1,090,961 in the 1–5 years interval (4221 ≥ 1 sepsis episode), and 1,339,201 in the 6–15 years interval (2771 ≥ 1 sepsis episode).

**Fig. 1:**
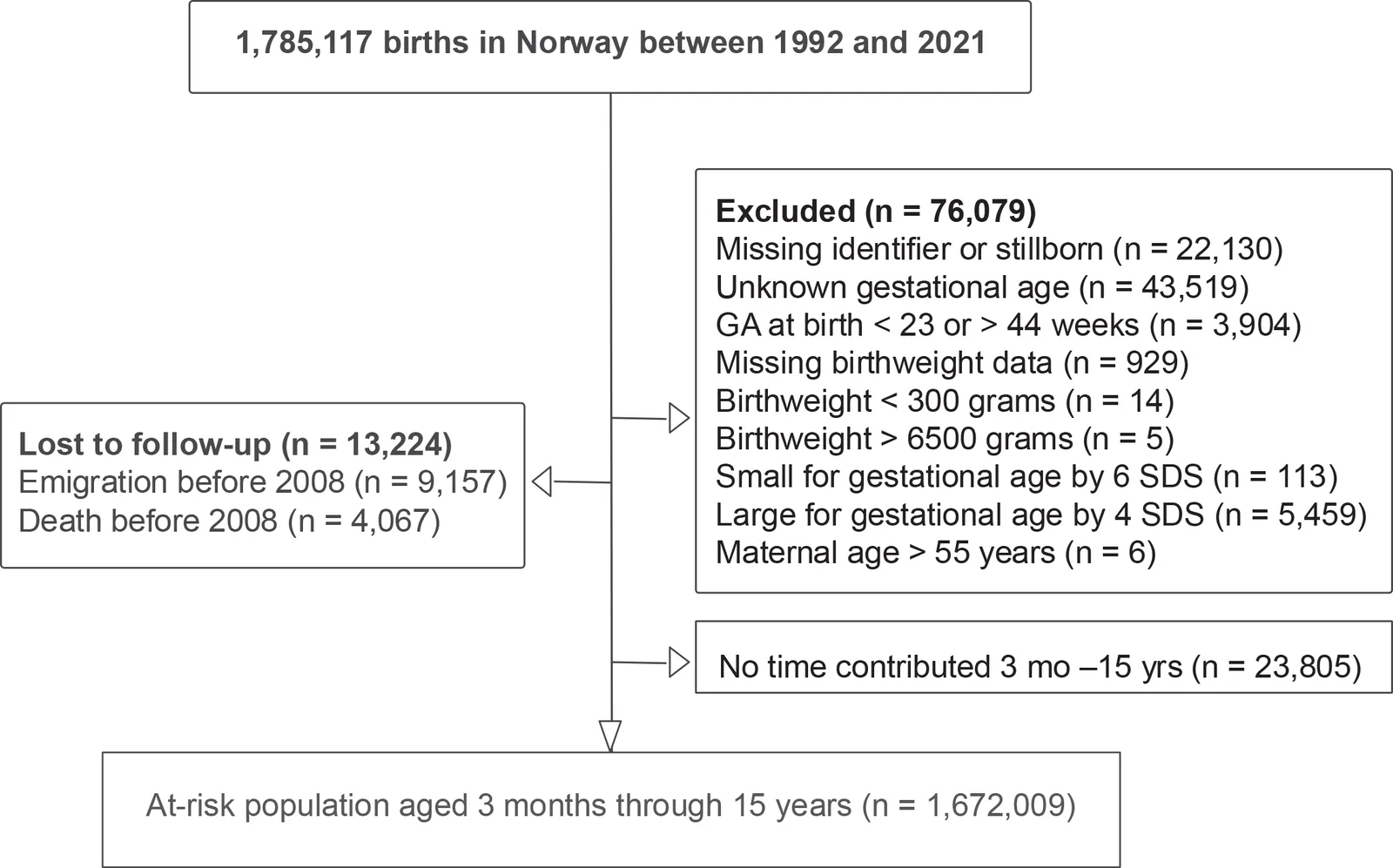
**Study profile.** After exclusions for missing or implausible values and losses to follow-up before 2008, a total of 1,672,009 children contributed person-time from age 3 months to 15 years during the period 2008–2021. Abbreviation: GA, gestational age.

**Fig. 2:**
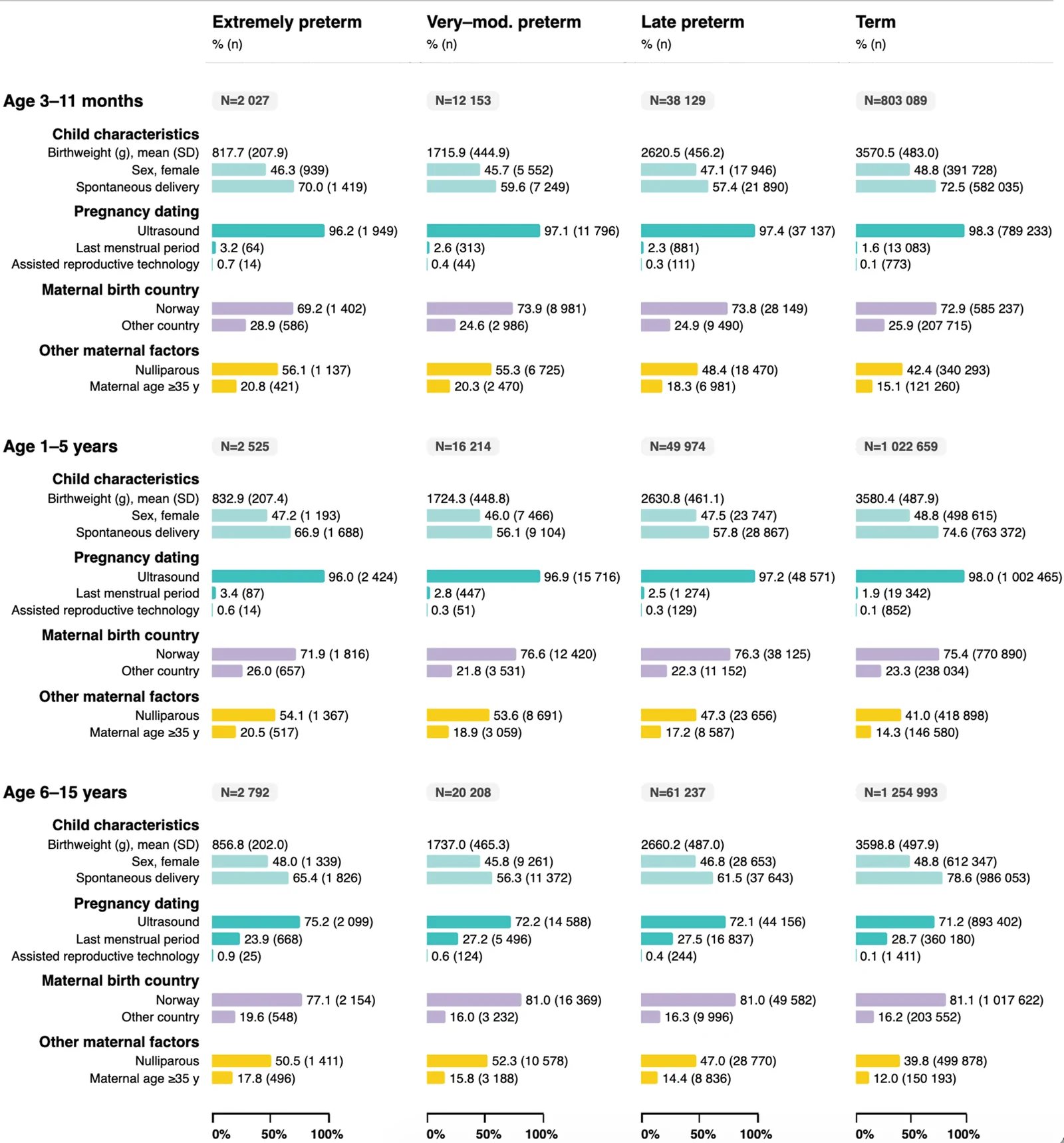
**Baseline characteristics of the at-risk population (2008**–**2021).** Distribution of child, maternal, and perinatal characteristics in the at-risk cohort (n = 1,672,009), summarised overall and by gestational-age categories (23–27, 28–33, 34–36, and 37–41 completed weeks). See Methods for variable definitions and data sources. Abbreviations: GA, gestational age; y, year(s).

Rates for sepsis-related admissions (implicit sepsis) were highest during infancy, particularly among infants born extremely preterm (23–27 weeks), with a rate of 84.4 per 1000 person-years (95% CI 62.2–106.7) at ages 3–11 months. In older children (6–15 years), full-term births (39–41 weeks) had the lowest rate at 0.6 per 1000 person-years (0.5–0.6; Fig. 3).

**Fig. 3:**
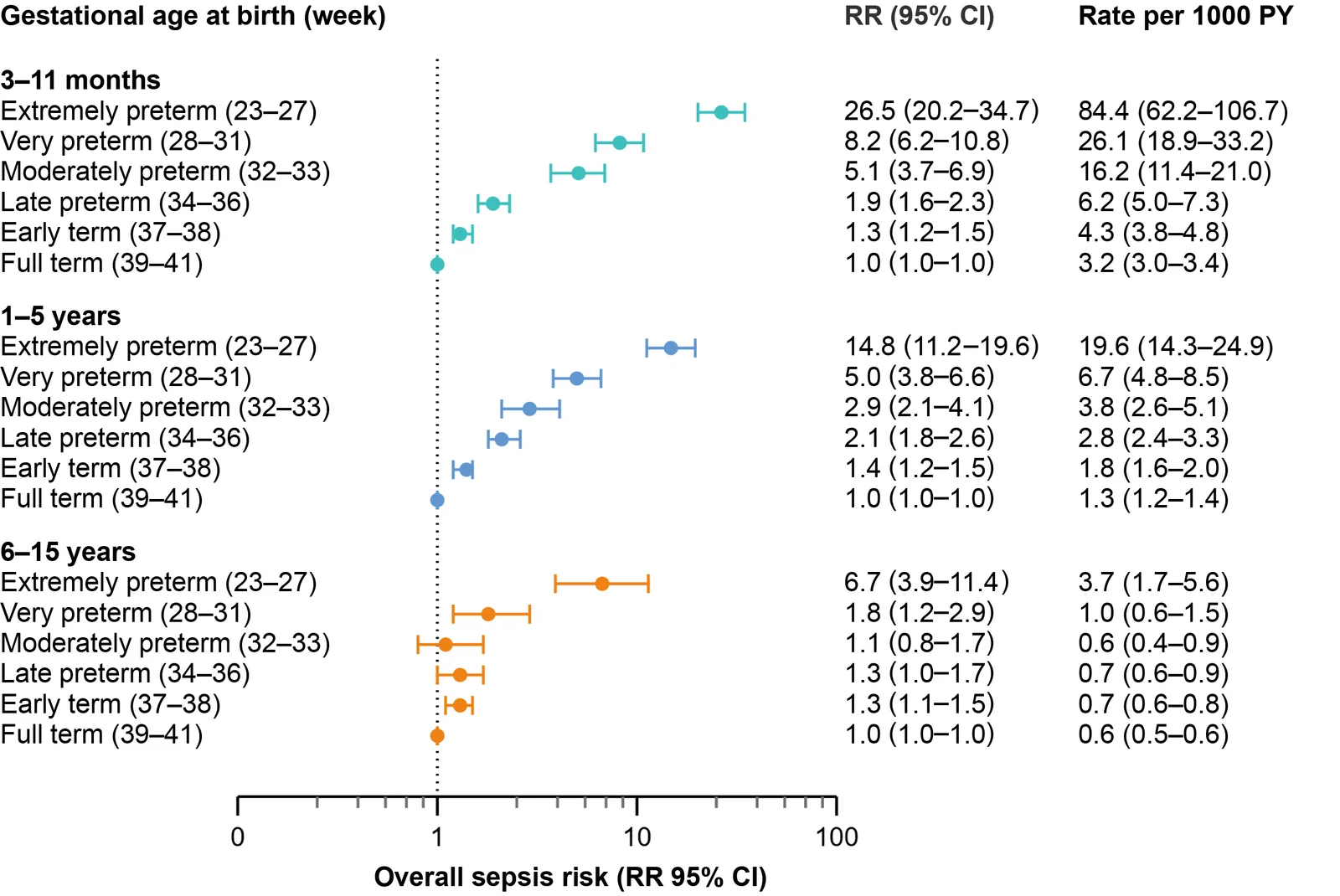
**Sepsis hospitalisation rates and rate ratios by gestational age.** Legend: Annual hospitalisation rates for all sepsis cases per 1000 person-years and rate ratios (RRs) with 95% CIs by gestational-age category across age strata (3–11 months, 1–5 years, 6–15 years), 2008–2021. RRs are shown on a logarithmic scale using 39–41 weeks as the reference category. Estimates are derived from negative binomial regression models; person-time excludes in-hospital days. Models include adjustment for covariates specified in Methods (e.g., birth year/month, sex, maternal age, parity, maternal immigration status, and GA ascertainment method). Abbreviations: GA, gestational age; RR, rate ratio; PY, person-years.

In infants aged 3–11 months, urinary tract infections were most frequent (944 episodes [37.3%]), followed by respiratory infections (757 [29.9%]) and multisystem infections (573 [22.8%]). Among children aged 1–5 years (n = 5670), respiratory infections predominated (1810 [31.9%]), followed by multisystem (1628 [28.7%]) and skin, soft tissue, and musculoskeletal infections (1339 [23.6%]); urinary tract infections occurred in 735 (12.9%). By ages 6–15 years, multisystem infections were most common (1642 [32.1%]), followed by skin, soft tissue, and musculoskeletal (1505 [23.4%]) and respiratory infections (949 [13.6%]). In older children, the aetiological spectrum broadened, and the proportion of unspecified sepsis decreased compared with younger age groups. Among cases with an explicit sepsis code, multisystem aetiology predominated across all age groups: 3–11 months (272 [72.1%]), 1–5 years (815 [72.2%]), and 6–15 years (883 [68.4%]) eFigure S1.

Across all age groups, infants born at earlier gestational ages had the highest hospitalisation rates for sepsis-related admissions (Fig. 3). At ages 3–11 months, the RR for sepsis-related hospitalisation among infants born very preterm compared with those born full-term was 8.2 (95% CI 6.2–10.8), whereas the RR for extremely preterm infants was 26.5 (95% CI 20.2–34.7). At ages 1–5 years, children born very preterm (28–31 weeks) remained at elevated risk of hospitalisation compared with full-term children, with an RR of 5.0 (95% CI 3.8–6.6).

Several sensitivity analyses were performed to assess the robustness of the findings. First, sibling comparison analyses were conducted (Fig. 4) to account for unmeasured familial confounding. These analyses demonstrated that children born at earlier gestational ages had a substantially increased risk of sepsis-related hospitalisation compared with their full-term siblings (39–41 weeks) across all age groups. For example, at ages 1–5 years, the OR for extremely preterm versus full-term siblings was 29.7 (95% CI 11.3–77.8). The patterns observed in sibling analyses were comparable to those in the primary population analyses, suggesting that the associations are unlikely to be fully explained by shared familial factors.

**Fig. 4:**
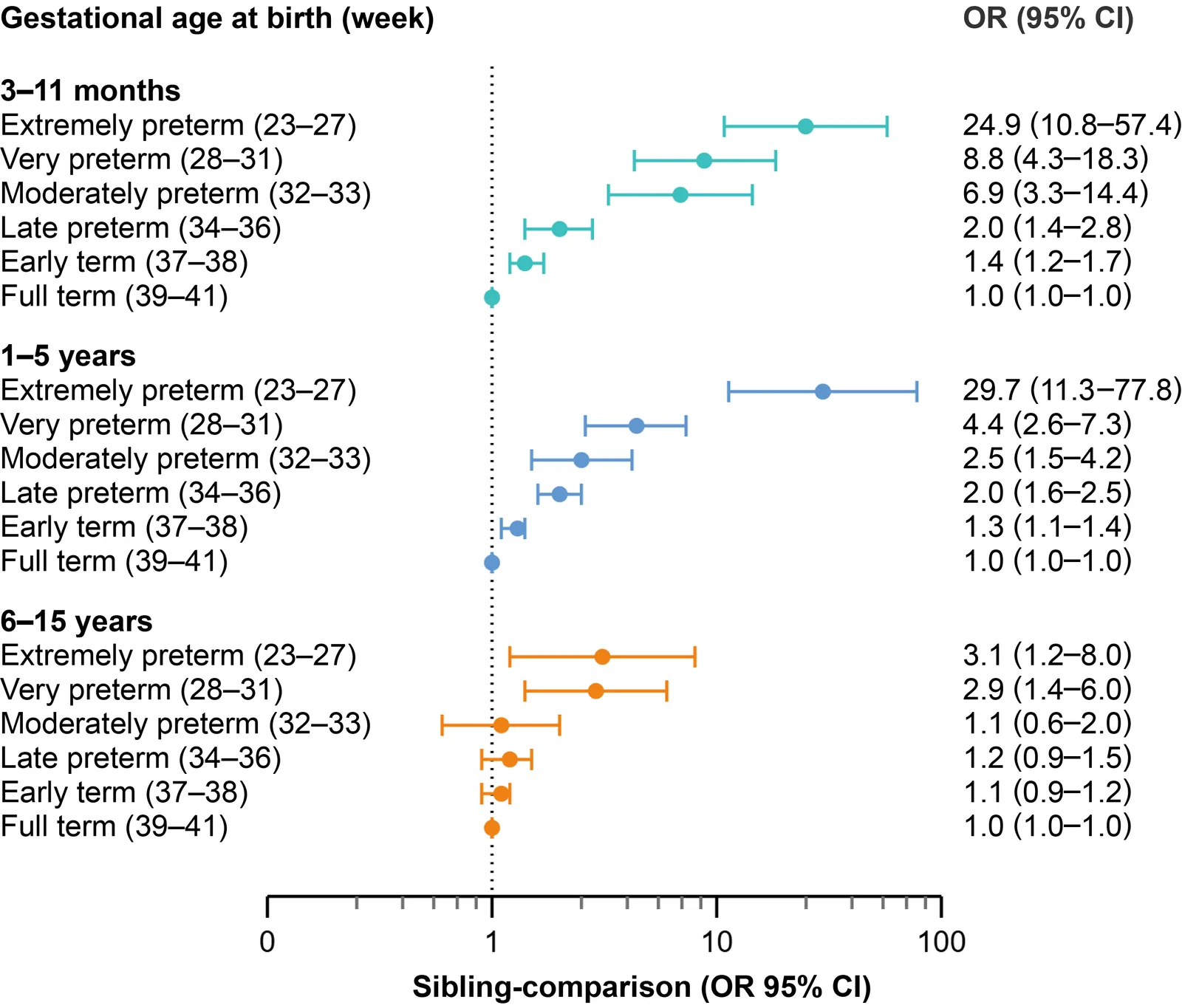
**Sibling-comparison estimates of sepsis risk by gestational age.** Within-family odds ratios (ORs) and 95% CIs are estimated using conditional logistic regression, comparing gestational age categories within maternal sibling sets contributing person-time in each age group (3–11 months, 1–5 years, and 6–15 years). The outcome is defined as ≥1 sepsis hospitalisation during the specified age interval. Models adjust for sex, birth month, birth year, maternal age, maternal birth country, parity, and GA ascertainment method, with mother-specific strata. Note that ORs from binary “≥1” outcomes are not directly comparable to the incidence-rate RRs presented in Fig. 3. Abbreviations: GA, gestational age; OR, odds ratio.

Second, a sensitivity analysis was conducted restricting the outcome strictly to explicit sepsis (Fig. 5). This analysis largely supported the findings from the main analysis, while less power yielded less precision for sepsis after infancy. The findings were somewhat less pronounced among young children aged 1–5 years, but the analysis otherwise reaffirmed that low gestational age remains a persistent risk factor for severe post-neonatal infections when using this stricter definition.

**Fig. 5:**
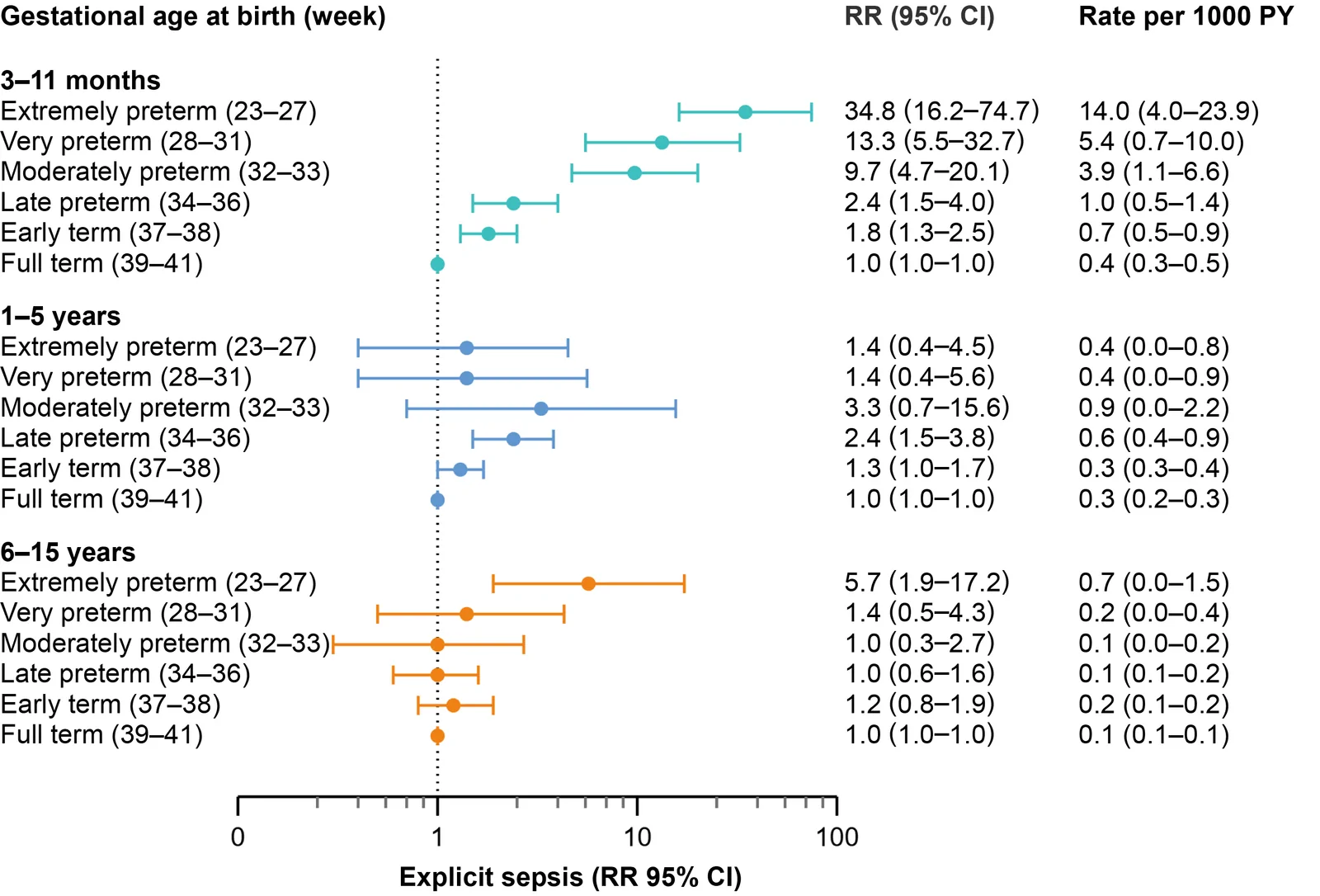
**Yearly hospitalisation rates per 1000 person-years and rate ratios (RRs) for explicit sepsis.** Yearly hospitalisation rates per 1000 person-years and RRs for explicit sepsis hospitalisations (2008–2021) are shown by gestational-age category at birth. RRs are plotted on a logarithmic scale, with individuals born at term (weeks 39–41) as the reference group. Error bars represent 95% CIs. Results are adjusted for year of birth, sex, birth month, parity, and maternal age.

Third, a pre-specified sensitivity analysis was conducted where respiratory failure was excluded as an organ dysfunction criterion for children hospitalised with respiratory infections (eFigure S1) to evaluate whether associations were driven primarily by severe respiratory conditions. At ages 3–11 months, the RR for extremely preterm children was 14.6 (95% CI 9.7–22.0). At ages 1–5 years, the RR for the extremely preterm group was attenuated but remained substantially elevated (RR 6.1, 95% CI 3.7–10.0).

Finally, analyses excluding medically indicated births were conducted to focus on spontaneous births and examine the potential impact of perinatal inflammatory processes underlying spontaneous birth (eFigure S2). Results were consistent with the main analyses. At 3–11 months, the RR for extremely preterm infants (23–27 weeks) was 22.6 (95% CI 13.8–37.0). For ages 1–5 years, the RR was 10.1 (95% CI 6.6–15.5), and for ages 6–15 years, the RR was 5.0 (95% CI 2.5–9.9). Results were similar in the children born after medically indicated deliveries (eFigure S3). In the 6–15-year age group, the rate ratios were similar to those in the main analysis; however, the CIs were wider, reflecting the smaller number of sepsis cases and the resulting reduced statistical precision in this restricted analysis. Finally, adjusting for birthweight Z-scores eFigure S5 or maternal education eFigure S6 and excluding children with congenital malformations (eFigure S7) showed results similar to the main analyses.

## Discussion

Our study demonstrates that shorter gestational age at birth is associated with an increased risk of sepsis-related hospitalisation, with risk progressively increasing as gestational age decreases, and that this pattern persists from early childhood through mid-adolescence. Notably, a dose–response relationship was observed between gestational age and sepsis-related hospitalisations, whereby even early-term births were associated with an elevated risk. These observations were supported by sensitivity analyses using a stricter definition for sepsis and using sibling comparison analyses.

Although no prior studies have specifically examined the risk of sepsis in preterm individuals during late childhood and adolescence, two recent studies have assessed hospitalisation risk for a broader range of infectious diseases.^3^, ^4^ A Norwegian study^3^ reported an elevated risk of hospitalisation due to infections among preterm individuals extending from childhood through adolescence, with a dose–response relationship that persists into adulthood. Conversely, a Danish study^26^ observed a decreased risk after 10–11 years of age; however, this finding was based on comparisons with children aged 2–3 years aged ≥37 weeks. The persistent uncertainty regarding infection risks during later childhood likely reflects the low incidence rate of severe infections in this age group. Previous studies indicate that low gestational age is a risk factor for a broad group of infectious diseases with multiple aetiologies, although there is evidence of a disproportionate vulnerability to RTIs.^3^, ^4^, ^27^ While acute respiratory failure may represent a systemic dysregulated response to infection, it may also be directly caused by RTI and not reflect true systemic sepsis. Recent trends toward increased use of implicit coding may reflect heightened awareness of the updated Sepsis-3 definition, which emphasises organ dysfunction.^11^, ^12^, ^28^, ^29^

The primary strength of this study is the use of comprehensive and nationwide registries covering 14 years, which enabled precise age-specific estimates of sepsis incidence across all gestational ages. We utilised both explicit diagnostic sepsis codes and a broader definition encompassing infection plus organ dysfunction, thereby reducing the impact of regional and temporal trends in sepsis coding. The robustness of our findings was further assessed by restricting analyses to spontaneous births and excluding cases involving respiratory failure. Additionally, sibling analyses controlled for shared familial confounding, albeit this approach may introduce bias from non-shared confounders.^30^ The consistency of results across these varied complementary approaches strengthens the validity of the findings. Although the national registries provide high population coverage and linkage errors are unlikely, misclassification of individual variables cannot be excluded. Gestational age is recorded in the Medical Birth Registry, whereas sepsis-related outcomes were identified from ICD-codes recorded during specialist healthcare. These codes may be affected by diagnostic and coding practices and could not be validated against medical records in this study. However, as the outcome represented severe infection requiring hospitalisation with organ dysfunction, clinically important events are likely to have been captured. Any remaining misclassification is expected to be largely non-differential by gestational age.

However, explicit sepsis codes may capture fewer organ-specific clinical presentations. When a diagnosis of sepsis is explicitly recorded, it describes a systemic condition in which a single organ focus may not be specified or may be less clinically prominent than the overall systemic response and thus associated with greater 28-day mortality.^31^ Explicit sepsis diagnosis codes have high specificity for clinically recognised sepsis (>90%), but at the cost of low sensitivity (<30%).^21^, ^22^ Furthermore, the permissive nature of the implicit case definition captures a broader group of severe infections requiring hospitalisation, which differs conceptually from modern, stringent physiological criteria such as the PHOENIX consensus definition.^12^ Nevertheless, our sensitivity analysis restricted to explicit sepsis only generally supported our main findings. Additionally, clinical awareness and ICD-10 coding practices for sepsis may have evolved over the 14-year study period.

Importantly, it is difficult to fully disentangle the independent biologic effect of prematurity from the downstream chronic medical conditions that often follow it, such as chronic lung disease, neurodisability, or cerebral palsy, which independently increase the risk of severe infection and lower the clinical threshold for hospitalisation. Therefore, our estimates reflect the total long-term association between prematurity and sepsis-related hospitalisation, inclusive of pathways mediated by these chronic conditions.

The primary limitations of this study are related to the use of administrative data to identify sepsis hospitalisations. Coding practices vary substantially with patient age and the cause of hospitalisation and have evolved. Consequently, reliance on strict criteria based solely on explicit sepsis diagnoses may introduce selection bias. Although combining explicit sepsis codes into a single outcome likely mitigates this challenge, some misclassification bias may persist, most likely biasing results toward the null. A second limitation is the differences in follow-up ages according to birth years, since the hospital diagnostic codes are only available from 2008. As a result, individuals born 1993–2002 did not contribute to the <6 years analyses, and those born after 2016 are not followed in adolescence, correspondingly (eFigure S8). Combined with low rates for sepsis hospitalisations in adolescents, the results for this group are less precise and should be interpreted with caution. Further,person-time calculation correctly excludes time after death from the at-risk period; however, it does not account for competing risks. This limitation is particularly relevant for the extremely preterm group, in which risk factors for death and sepsis may overlap, potentially leading to an underestimation of incidence rates.^32^ Moreover, because many infants in this age group may have been hospitalised since birth, including the neonatal period, this could result in an underestimation of the true incidence of community-acquired sepsis. We did not have access to detailed neonatal data and could not separately assess the role of neonatal sepsis in later sepsis risk. Nevertheless, the risk of sepsis in the neonatal period is well-established in previous studies, and our previous work addressed this age group separately by excluding diagnoses specifically related to the neonatal period (e.g., ICD-10 code P36 for bacterial sepsis of newborns).^33^

Our findings indicate that the increased susceptibility to severe infections requiring hospitalisation in individuals born preterm is not confined to the neonatal period but represents a persistent vulnerability extending into later childhood and adolescence. Several interrelated mechanisms related to underlying alterations in immune development and microbiome, alongside the potential consequences of a higher comorbidity burden may contribute to this pattern. Preterm birth occurs during a critical period of immune and microbiome maturation, and disruption of these developmental processes may have lasting effects on immune regulation, epithelial barrier function, and responses to infection.^34^ Delayed organ development, small organ size, and an elevated risk of chronic disease development, such as chronic respiratory diseases,^24^ along with a higher prevalence of severe disabilities, including cerebral palsy, likely contribute, at least in part, to the increased risk of severe infections. However, these biological hypotheses must be weighed against non-biological explanations, such as greater clinical surveillance or lower admission thresholds for medically complex children. Immunological characteristics, perinatal pathogen exposure, and increased hospital contact may contribute to this pattern, although attributing risk to individual factors or immune functions is challenging.^35^ As the present study cannot attribute the observed associations to individual pathways, future research should aim to elucidate the downstream mechanisms and determinants that contribute to heightened sepsis susceptibility in preterm-born children. Improved understanding of these processes could inform the development of interventions aimed at reducing susceptibility to sepsis and immune-mediated disorders.

Effective strategies to prevent post-neonatal infections in preterm-born children remain limited. Few studies have evaluated appropriate non-pharmaceutical infection prevention programs that may benefit preterm-born children.^36^ Although childhood immunisations are well documented to be safe and effective in preterm individuals,^37^ their implementation is often hindered, as vaccination coverage among preterm-born children tends to be lower than among those born at term.^38^ Moreover, evidence on the effectiveness of newer and risk-based vaccines in this population is limited, and gestational age is often not considered in recommendations targeting high-risk children. Given the persisting susceptibility to severe infections requiring hospitalisation, implementing prevention and treatment strategies that account for this elevated risk could help reduce the lifelong burden of severe infectious diseases in individuals born preterm.

In this nationwide registry-based study, shorter gestational age at birth was associated with an increased risk of sepsis-related hospitalisations from infancy through mid-adolescence, with stronger associations at lower gestational ages. Although the exact impact of premature birth on intrinsic immune function versus chronic comorbidity burden remains incompletely understood, it is relevant to consider gestational age at birth when assessing risk of childhood severe infections.

## Contributors

JV, AA, and KR directly accessed and verified the underlying data reported in the manuscript at the level of the individual participant. All authors had full access to all the data in the study and accept responsibility to submit for publication. JV, SMN, TR, ES, and KR conceptualised and designed the study. All authors contributed to the acquisition, analysis, or interpretation of data. JV drafted the initial manuscript, with SMN and KR contributing to the drafting. All authors critically revised the manuscript for important intellectual content. JV, AA, and TR performed the statistical analysis. KR, SMN, and TR supervised the study. All authors take final responsibility for the decision to submit the report for publication.

## Data sharing statement

Deidentified individual-level registry data used in this study cannot be shared publicly due to legal restrictions of the Medical Birth Registry of Norway and the Norwegian Patient Registry. Data access may be requested directly from the registry holders subject to Norwegian regulations and approvals. The analysis code and variable metadata is available at GitHub (https://github.com/andreasasheim/Sepsis_PT) prior to publication and at request to the corresponding author.

## Declaration of interests

The authors declare no competing interests.
