# Supplementary material for: Sepsis-related hospitalisation risk in preterm-born and term-born children and adolescents: a nationwide registry study
Source: eClinicalMedicine. 2026 Nov 3;101:104295. doi: 10.1016/j.eclinm.2026.104295 (PMC13756306; doi:10.1016/j.eclinm.2026.104295)
Supplement: Supplementary file 1 [file mmc1.docx]

**Online-Only Supplements**

**eFigure 1.** Sensitivity analysis of sepsis rate ratios (RR) for excluding respiratory failure.

Legend: RRs for sepsis are shown after excluding respiratory failure as a contributing qualifier in patients hospitalized with respiratory tract infections. The analysis stratifies risk by gestational-age category and age group. Error bars represent 95% confidence intervals (95% CI). Abbreviations: CI, confidence interval; GA, gestational age; RR, rate ratio; PY, person-years.

**eFigure 2:** Sensitivity analysis for medically indicated births.

Legend: Adjusted rate ratios (95% CIs) for all sepsis are shown by gestational age category for medically indicated births (e.g., inductions or planned cesarean deliveries), stratified by age group (3–11 months, 1–5 years, 6–15 years). The reference category is 39–41 weeks. Models include adjustments for covariates as in Figure 3. Abbreviations: CI, confidence interval; GA, gestational age; RR, rate ratio; PY, person-years.

**eFigure 3:** Sensitivity analysis for spontaneous births.

Legend: Adjusted rate ratios (95% CIs) for all sepsis are shown by gestational age category for spontaneous births, stratified by age group (3–11 months, 1–5 years, 6–15 years). The reference category is 39–41 weeks. Models include adjustments for covariates as in Figure 3. Abbreviations: CI, confidence interval; GA, gestational age; RR, rate ratio; PY, person-years.

**eFigure 4.** The age of participants by birth year and follow-up.

All sepsis:

Explicit sepsis:

**eFigure 5:** Sepsis etiology by organ system.

Legend: Sepsis etiology is shown by organ system, stratified by gestational-age category and follow-up age group.

**eFigure 6:** Sensitivity analysis of sepsis rate ratios (RR), adjusted for z-score.

Legend: Adjusted rate ratios (95% CIs) for all sepsis are shown by gestational age category adjusted for z-score, stratified by age group (3–11 months, 1–5 years, 6–15 years). The reference category is 39–41 weeks. Models include adjustments for covariates as in Figure 3. Abbreviations: CI, confidence interval; GA, gestational age; RR, rate ratio; PY, person-years.

**eFigure 7:** Sensitivity analysis of sepsis rate ratios (RR), adjusting for maternal education.

Legend: Adjusted rate ratios (95% CIs) for all sepsis are shown by gestational age category adjusted for maternal education, stratified by age group (3–11 months, 1–5 years, 6–15 years). The reference category is 39–41 weeks. Models include adjustments for covariates as in Figure 3. Abbreviations: CI, confidence interval; GA, gestational age; RR, rate ratio; PY, person-years.

**eFigure 8:** Sensitivity analysis of sepsis rate ratios (RR), excluding children with congenital malformations.

Legend: Adjusted rate ratios (95% CIs) for all sepsis are shown by gestational age category, excluding congenital malformations, stratified by age group (3–11 months, 1–5 years, 6–15 years). The reference category is 39–41 weeks. Models include adjustments for covariates as in Figure 3. Abbreviations: CI, confidence interval; GA, gestational age; RR, rate ratio; PY, person-years.

**eTable 1:** ICD-10 Codes for Explicit and Implicit Sepsis and Associated Organ Dysfunction

| ICD-10 code | | Explicit sepsis | Implicit sepsis | Organ dysfunction |
| --- | --- | --- | --- | --- |
| Chapter I: Certain infectious and parasitic diseases (A00-B99) | |  |  |  |
| A00–A09 | Intestinal infectious diseases |  | ● |  |
| A19 | Acute miliary tuberculosis |  | ● |  |
| A20 | Plague |  | ● |  |
| A20.7 | Septicaemic plague | ● |  |  |
| A21 | Tularaemia |  | ● |  |
| A21.7 | Generalized tularaemia | ● |  |  |
| A22 | Anthrax |  | ● |  |
| A22.7 | Anthrax sepsis | ● |  |  |
| A23 | Brucellosis |  | ● |  |
| A24 | Glanders and melioidosis |  | ● |  |
| A24.1 | Acute and fulminating melioidosis | ● |  |  |
| A25 | Rat-bite fevers |  | ● |  |
| A26 | Erysipeloid |  | ● |  |
| A26.7 | Erysipelothrix sepsis | ● |  |  |
| A27 | Leptospirosis |  | ● |  |
| A28 | Other zoonotic bacterial diseases, not elsewhere classified |  | ● |  |
| A28.2 | Extraintestinal yersiniosis | ● |  |  |
| A30-A32 | Certain bacterial diseases |  | ● |  |
| A32.7 | Listerial sepsis | ● |  |  |
| A36 | Diphtheria |  | ● |  |
| A37 | Whooping cough |  | ● |  |
| A38 | Scarlet fever |  | ● |  |
| A39 | Meningococcal infection |  | ● |  |
| A39.4 | Meningococcaemia, unspecified | ● |  |  |
| A40 | Streptococcal sepsis | ● |  |  |
| A41 | Other sepsis | ● |  |  |
| A42 | Actinomycosis |  | ● |  |
| A42.7 | Actinomycotic sepsis | ● |  |  |
| A43 | Nocardiosis |  | ● |  |
| A44 | Bartonellosis |  | ● |  |
| A46 | Erysipelas |  | ● |  |
| A48 | Other bacterial diseases, not elsewhere classified |  | ● |  |
| A48.3 | Toxic shock syndrome |  |  | ● |
| A49 | Bacterial infection of unspecified site |  | ● |  |
| A54 | Gonococcal infection |  | ● |  |
| A54.8 | Other gonococcal infections (incl. sepsis) |  |  |  |
| A54.86 | Gonococcal sepsis (country-specific subcode) | ● |  |  |
| A59 | Trichomoniasis |  | ● |  |
| A69.0-A69.1 | Necrotizing ulcerative stomatitis/Other Vincent's infections |  | ● |  |
| A69.9 | Spirochaetal infection, unspecified |  | ● |  |
| A70 | Chlamydia psittaci infection |  | ● |  |
| A74 | Other diseases caused by chlamydiae |  | ● |  |
| A75-A79 | Rickettsioses |  | ● |  |
| A80-A89 | Viral infections of the central nervous system |  | ● |  |
| A90-A99 | Arthropod-borne viral fevers and viral haemorrhagic fevers |  | ● |  |
| B00 | Herpesviral [herpes simplex] infections |  | ● |  |
| B00.7 | Disseminated herpesviral disease | ● |  |  |
| B01-B09 | Viral infections characterized by skin and mucous membrane lesions |  | ● |  |
| B25-B27 | Other herpesviral infections |  | ● |  |
| B33-B34 | Other viral diseases, not elsewhere classified |  | ● |  |
| B37 | Candidiasis |  | ● |  |
| B37.7 | Candidal sepsis | ● |  |  |
| B38-B49 | Mycoses |  | ● |  |
| B50-B64 | Protozoal diseases |  | ● |  |
| B67 | Echinococcosis |  | ● |  |
| B95-B97 | Bacterial, viral and other infectious agents |  | ● |  |
| B99 | Other and unspecified infectious diseases |  | ● |  |
| Chapter III: Diseases of the blood and blood-forming organs (D50-D89) | |  |  |  |
| D69.5 | Secondary thrombocytopenia |  |  | ● |
| Chapter IV: Endocrine, nutritional and metabolic diseases (E00-E90) | |  |  |  |
| E87.2 | Acidosis |  |  | ● |
| Chapter VI: Diseases of the nervous system (G00-G99) | |  |  |  |
| G00-G08 | Inflammatory diseases of the central nervous system |  | ● |  |
| G93.4 | Encephalopathy, unspecified |  |  | ● |
| Chapter VII: Diseases of the eye and adnexa (H00-H59) | |  |  |  |
| H05.0 | Acute inflammation of orbit |  | ● |  |
| H60.2 | Malignant otitis externa |  | ● |  |
| H70.0 | Acute mastoiditis |  | ● |  |
| Chapter IX: Diseases of the circulatory system (I00-I99) | |  |  |  |
| I00 | Rheumatic fever without mention of heart involvement |  | ● |  |
| I26.0 | Pulmonary embolism with mention of acute cor pulmonale |  | ● |  |
| I26.9 | Pulmonary embolism without mention of acute cor pulmonale |  | ● |  |
| I33 | Acute and subacute endocarditis |  | ● |  |
| I33.0 | Acute and subacute infective endocarditis |  |  | ● |
| I33.9 | Acute endocarditis, unspecified |  |  | ● |
| I38 | Endocarditis, valve unspecified |  | ● |  |
| I39 | Endocarditis and heart valve disorders in diseases classified elsewhere |  | ● |  |
| I40.0 | Infective myocarditis |  | ● |  |
| I46 | Cardiac arrest |  |  | ● |
| I76 | Umbilical artery embolism and thrombosis |  | ● |  |
| I95.9 | Hypotension, unspecified |  |  | ● |
| I96 | Gangrene, not elsewhere classified |  | ● |  |
| Chapter X: Diseases of the respiratory system (J00-J99) | |  |  |  |
| J01-J06 | Acute upper respiratory infections |  | ● |  |
| J09-J18 | Influenza and pneumonia |  | ● |  |
| J20-J22 | Other acute lower respiratory infections |  | ● |  |
| J36 | Peritonsillar abscess |  | ● |  |
| J39.0-J39.1 | Retropharyngeal and parapharyngeal abscess/Other abscess of pharynx |  | ● |  |
| J80 | Acute respiratory distress syndrome |  |  | ● |
| J85-J86 | Suppurative and necrotic conditions of the lower respiratory tract |  | ● |  |
| J95.2 | Acute pulmonary insufficiency following nonthoracic surgery |  |  | ● |
| J96 | Respiratory failure, not elsewhere classified |  |  | ● |
| J96.0 | Acute respiratory failure |  |  | ● |
| J96.2 | Acute on chronic respiratory failure |  |  | ● |
| J96.9 | Respiratory failure, unspecified |  |  | ● |
| Chapter XI: Diseases of the digestive system (K00-K93) | |  |  |  |
| K35-K37 | Appendicitis |  | ● |  |
| K57 | Diverticular disease of intestine |  | ● |  |
| K61 | Abscess of anal and rectal regions |  | ● |  |
| K63.0-K63.1 | Abscess of intestine/Perforation of intestine (nontraumatic) |  | ● |  |
| K65 | Peritonitis |  | ● |  |
| K72.0 | Acute and subacute hepatic failure |  |  | ● |
| K72.9 | Hepatic failure, unspecified |  |  | ● |
| K75.0 | Abscess of liver |  | ● |  |
| K81.0 | Acute cholecystitis |  | ● |  |
| K81.2 | Acute cholecystitis with perforation |  | ● |  |
| K83.0 | Cholangitis |  | ● |  |
| K95.0 | Infection of gastrostomy or enterostomy |  | ● |  |
| K95.8 | Other complications of gastrostomy or enterostomy |  | ● |  |
| Chapter XII: Diseases of the skin and subcutaneous tissue (L00-L99) | |  |  |  |
| L02-L04 | Cutaneous abscess, furuncle and carbuncle/Cellulitis/Acute lymphadenitis |  | ● |  |
| L08 | Other local infections of skin and subcutaneous tissue |  | ● |  |
| Chapter XIII: Diseases of the musculoskeletal system and connective tissue (M00-M99) | |  |  |  |
| M00 | Pyogenic arthritis |  | ● | ● |
| M01 | Direct infections of joint in infectious and parasitic diseases |  | ● |  |
| M01.3 | Arthritis in other bacterial diseases classified elsewhere |  |  | ● |
| M72.6 | Necrotizing fasciitis |  |  | ● |
| M86 | Osteomyelitis |  | ● |  |
| M86.0 | Acute haematogenous osteomyelitis |  |  | ● |
| M86.1 | Other acute osteomyelitis |  |  | ● |
| M86.2 | Subacute osteomyelitis |  |  | ● |
| M86.9 | Osteomyelitis, unspecified |  |  | ● |
| Chapter XIV: Diseases of the genitourinary system (N00-N99) | |  |  |  |
| N00 | Acute nephritic syndrome |  |  | ● |
| N10 | Acute tubulo-interstitial nephritis |  | ● |  |
| N15.1 | Renal and perinephric abscess |  | ● |  |
| N17 | Acute kidney failure |  |  | ● |
| N30 | Cystitis |  | ● |  |
| N39.0 | Urinary tract infection, site not specified |  | ● |  |
| N41.0 | Acute prostatitis |  | ● |  |
| N41.2-N41.3 | Abscess of prostate/Prostatocystitis |  | ● |  |
| N45 | Orchitis and epididymitis |  | ● |  |
| N70-N74 | Inflammatory diseases of female pelvic organs |  | ● |  |
| N98.0 | Infection associated with artificial insemination |  | ● |  |
| Chapter XV: Pregnancy, childbirth and the puerperium (O00-O99) | |  |  |  |
| O03.0, O03.5 | Genital tract and pelvic infection following spontaneous abortion... |  | ● |  |
| O03.8 | Other specified complications following spontaneous abortion... |  | ● |  |
| O04.5, O04.8 | Genital tract and pelvic infection following medical abortion... |  | ● |  |
| O07.3 | Genital tract and pelvic infection following other or unspecified abortion |  | ● |  |
| O08.0 | Genital tract and pelvic infection following abortion... |  | ● |  |
| O08.8 | Other specified complications following abortion... |  | ● |  |
| O23 | Genitourinary tract infection in pregnancy |  | ● |  |
| O75.3 | Other infection during labour |  | ● |  |
| O85 | Puerperal sepsis | ● |  |  |
| O86 | Other puerperal infections |  | ● |  |
| O88.3 | Obstetric pyemic and septic embolism |  | ● |  |
| O91 | Infections of breast associated with childbirth |  | ● |  |
| O98 | Maternal infectious and parasitic diseases classified elsewhere... |  | ● |  |
| Chapter XVI: Certain conditions originating in the perinatal period (P00-P96) | |  |  |  |
| P36 | Bacterial sepsis of newborn | ● |  |  |
| Chapter XVIII: Symptoms, signs and abnormal clinical and laboratory findings (R00-R99) | |  |  |  |
| R09.2 | Respiratory arrest |  |  | ● |
| R40.0 | Somnolence |  |  | ● |
| R40.1 | Stupor |  |  | ● |
| R40.2 | Coma, unspecified |  |  | ● |
| R41.8 | Other and unspecified symptoms and signs involving cognitive functions |  |  | ● |
| R55 | Syncope and collapse |  |  | ● |
| R57 | Shock, not elsewhere classified |  |  | ● |
| R57.2 | Septic shock | ● |  |  |
| R65.1 | Systemic inflammatory response syndrome (SIRS) of infectious origin with organ failure | ● |  |  |
| R78.8 | Finding of other specified drugs and other substances... |  |  |  |
| R78.81 | Bacteremia (country-specific subcode) |  | ● |  |
| Chapter XIX: Injury, poisoning and certain other consequences of external causes (S00-T98) | |  |  |  |
| T80.2 | Infections following infusion, transfusion and therapeutic injection |  | ● |  |
| T81.4 | Infection following a procedure, not elsewhere classified |  | ● |  |
| T82.6-T82.7 | Infection and inflammatory reaction due to cardiac and vascular devices... |  | ● |  |
| T83.5-T83.6 | Infection and inflammatory reaction due to prosthetic device... in urinary system |  | ● |  |
| T84.5-T84.7 | Infection and inflammatory reaction due to internal joint prosthesis/internal fixation device |  | ● |  |
| T85.7 | Infection and inflammatory reaction due to other internal prosthetic devices... |  | ● |  |
| T88.0 | Infection following immunization |  | ● |  |
| Chapter XXII: Codes for special purposes (U00-U49) | |  |  |  |
| U04 | Severe acute respiratory syndrome [SARS] |  | ● |  |

**eAppendix 1**: Excerpts from the study protocol submitted to the funding agency (May 2024)
